# Gut colonization and subsequent infection of neonates caused by extended-spectrum beta-lactamase-producing *Escherichia coli* and *Klebsiella pneumoniae*

**DOI:** 10.3389/fcimb.2023.1322874

**Published:** 2024-01-19

**Authors:** Verónica Jiménez-Rojas, Dina Villanueva-García, Ana Luisa Miranda-Vega, Rubén Aldana-Vergara, Pamela Aguilar-Rodea, Beatriz López-Marceliano, Alfonso Reyes-López, María Dolores Alcántar-Curiel

**Affiliations:** ^1^ Unidad de Investigación en Enfermedades Infecciosas, Hospital Infantil de México Federico Gómez, Ciudad de México, Mexico; ^2^ Departamento de Neonatología, Hospital Infantil de México Federico Gómez, Ciudad de México, Mexico; ^3^ Centro de Estudios Económicos y Sociales en Salud, Hospital Infantil de México Federico Gómez, Ciudad de México, Mexico; ^4^ Laboratorio de Infectología, Microbiología e Inmunología Clínica. Unidad de Investigación en Medicina Experimental, Facultad de Medicina, Universidad Nacional Autónoma de México, Ciudad de México, Mexico

**Keywords:** *Escherichia coli*, *Klebsiella pneumoniae*, ESBL - extended-spectrum beta-lactamase, gut colonization, HAI - healthcare-associated infection, neonates

## Abstract

The gut microbiota harbors diverse bacteria considered reservoirs for antimicrobial resistance genes. The global emergence of extended-spectrum beta-lactamase (ESBL)-producing Enterobacterales (ESBL-PE) significantly contributes to healthcare-associated infections (HAIs). We investigated the presence of ESBL-producing *Escherichia coli* (ESBL-PEco) and ESBL-producing *Klebsiella pneumoniae* (ESBL-PKpn) in neonatal patients’ guts. Furthermore, we identified the factors contributing to the transition towards ESBL-PEco and ESBL-PKpn-associated healthcare-associated infections (HAIs). The study was conducted from August 2019 to February 2020, in a Neonatal Intensive Care Unit of the Hospital Infantil de México Federico Gómez. Rectal samples were obtained upon admission, on a weekly basis for a month, and then biweekly until discharge from the neonatology ward. Clinical data, culture results, and infection information were gathered. We conducted antimicrobial tests, multiplex PCR assay, and pulsed-field gel electrophoresis (PFGE) to determine the antimicrobial resistance profile and genetic relationships. A comparison between the group’s controls and cases was performed using the Wilcoxon and Student t-tests. Of the 61 patients enrolled, 47 were included, and 203 rectal samples were collected, identifying 242 isolates. In 41/47 (87%) patients, colonization was due to ESBL-PEco or ESBL-PKpn. And nine of them developed HAIs (22%, 9/41). ESBL-PEco resistance to cephalosporins ranged from 25.4% to 100%, while ESBL-PKpn resistance varied from 3% to 99%, and both bacteria were susceptible to carbapenems, tigecillin, and colistin. The prevalent *bla*_CTX-M-group-1_ gene accounted for 77.2% in ESBL-PEco and 82.2% in ESBL-PKpn, followed by *bla*_TEM_ 50% and *bla*_OXA-1_ 43.8% in ESBL-PEco and *bla*_TEM_ 80.2% and *bla*_SHV_ 76.2% in ESBL-PKpn. Analysis of clonality revealed identical colonizing and infection isolates in only seven patients. Significant risk factors included hospital stay duration, duration of antibiotic treatment, and invasive device usage. Our findings suggest high ESBL-PEco and ESBL-PKpn rates of colonization often lead to infection in neonates. Attention should be paid to patients with ESBL-PE.

## Introduction

Immediately following birth, the establishment of microorganisms begins, shaping the microbiota of newborns (Groer et al., 2014). The composition of this microbiota is influenced by various factors, including the mode of delivery, gestational age, birth weight, among others (Collado et al., 2012; Groer et al., 2014; Prince et al., 2014).

Unfortunately, hospitalized newborns are exposed and vulnerable to acquiring an altered microbiome due to the atypical care environment they experience in a Neonatal Intensive Care Unit (NICU) (Hartz et al., 2015). Coupled with their nascent immune system and multiple invasive procedures, they become susceptible to healthcare-associated infections (HAIs) (Calva et al., 1996; Groer et al., 2014). A significant influencing factor is medical treatment, particularly antibiotics, which have led to the emergence of bacteria capable of evading their action through various resistance mechanisms (Bäckhed et al., 2012; Vangay et al., 2015). Notably, among these resistant strains are ESBL-producing *Escherichia coli* (ESBL-PEco) and ESBL-producing *Klebsiella pneumoniae* (ESBL-PKpn), these Gram-negative rod-shaped bacteria are classified within the Enterobacteriaceae family of the order Enterobacterales. They are responsible for a wide array of infections that exacerbate healthcare challenges due to their remarkable ability to develop resistance against multiple drugs, severely limiting available treatment options. Consequently, the World Health Organization (WHO) has categorized them as “pathogens with critical priority” due to their escalating prevalence as causative agents of HAIs, underscoring the urgent need for research and the development of novel antibiotics (Tacconelli et al., 2018; CDC, 2019).

The presence of ESBL-PE in the gut microbiota could be a risk to both colonized patients and those who share the hospital environment (Armand-Lefèvre et al., 2018; Souverein et al., 2019; Denkel et al., 2020; Campos-Madueno et al., 2023).

The aim of this study was to prospectively investigate the incidence of neonates intestinally colonized with ESBL-PEco or ESBL-PKpn and the factors enabling the progression from colonization to infectious disease. Furthermore, we sought to characterize the ESBL genes and the clonality of the isolates.

## Materials and methods

### Study design, period, and area

A prospective case-control study was conducted between August 1, 2019, and February 28, 2020, in patients of both genders admitted to the NICU of the Federico Gómez Children’s Hospital (HIMFG), a tertiary pediatric hospital in Mexico City, Mexico. The hospital has 229 beds across various specialties, with 30 beds specifically located in the neonatology hospitalization area. The Research, Ethics, and Biosafety Committees evaluated the study design and data sources for the HIM/2018/073, SSA 1522 study protocol, which involved a review of medical records generated during routine patient care. This study did not involve the performance of medical intervention or intentional modification of physiological variables by their searcher on patients or their legal guardians. Therefore, this project was classified as risk-free research for the study subjects. The informed consent emphasized the researchers’ obligation to protect the identity and privacy of the patients included in the study.

### Population, sample size, and data collection

After informed consent was obtained, rectal swabs were collected from all newborns admitted to the NICU for active surveillance and purposeful search of colonizing extended-spectrum beta-lactamases-producing ESBL-PEco and ESBL-PKpn. Demographic and clinical information of the patients was collected. Subsequently, rectal samples were collected weekly until the patients completed one month of hospitalization. Afterward, samples were collected every two weeks until discharge from the neonatology ward. All patient-related healthcare-associated infections (HAIs) were prospectively followed. HAIs were classified if the isolate was obtained after 48 h of NICU admission. When a patient presented an infectious event, the cultures identified as causing HAIs and the clinical data were collected.

### Sample processing, bacterial isolation, and ESBL-PEco and ESBL-PKpn detection

Rectal samples were obtained using cotton swabs soaked in Cary-Blair transport medium, followed by inoculation and overnight culturing on MacConkey and 5% sheep blood agar plates. Subsequently, distinctive colonies of *E. coli* and *K. pneumoniae* were chosen and transferred to the VITEK 2 AST automated system (bioMérieux, Marcy-l’Étoile, France) for bacterial identification (with an accuracy range of 98-99% for *E. coli* and 95-97% for *K. pneumoniae*) and antimicrobial susceptibility testing. Strains from infectious processes were subculture on 5% sheep blood agar and incubated at 37°C for 18-24 h. The antibiotics evaluated included amikacin (AN), ampicillin-sulbactam (AMS), cefepime (FEP), cefoxitin (FOX), ceftazidime (CAZ), ceftriaxone (CRO), ciprofloxacin (CIP), colistin (COL), doripenem (DOR), ertapenem (ETP), gentamicin (GM), imipenem (IPM), meropenem (MEM), piperacillin/tazobactam (TZP), and tigecycline (TGC). Antibiotic susceptibility was interpreted using the Clinical and Laboratory Standards Institute (CLSI) guidelines (CLSI, 2023), and ESBL categorization was determined based on the susceptibility pattern to different cephalosporins.

The confirmation of ESBL-producing isolates was conducted using the combination disc method. A comparison of the zone of inhibition was made between ceftazidime (30 µg) and cefotaxime (30 µg) discs alone versus those of ceftazidime and cefotaxime discs containing clavulanic acid (10 µg). Following the incubation period, isolates displaying an increase in the zone diameter of ≥5 mm around either of the clavulanate combined discs compared to that of the disc alone were considered ESBL producers, according the criteria recommended by CLSI (CLSI, 2023). *E. coli* ATCC 25922 and *K. pneumoniae* ATCC 700603 strains were used as negative and positive controls for ESBL production, respectively.

### ESBL gene detection

The total genomic DNA was extracted from an isolated colony of the strains using the Wizard Genomic DNA Purification kit (Promega, Madison, WI) following the manufacturer’s instructions. The ESBL genes were detected using specific primer sets in PCR assays as previously described (Dallenne et al., 2010). The first assay involved a multiplex PCR targeting *bla*_TEM_, *bla*_SHV_, and *bla*_OXA-1-like_ genes, while the second assay focused on *bla*_CTX-M_ genes, covering phylogenetic groups 1, 2, and 9. Within these groups, specific variants were probed, including CTX-M-1, CTX-M-3, and CTX-M-15 from CTX-M group 1, CTX-M-2 from CTX-M group 2, and CTX-M-9, CTX-M-14 from CTX-M group 9. The third assay utilized simplex PCR for *bla*_CTX-M-8/25_ groups, detecting CTX-M-8, CTX-M-25, CTX-M-26, and CTX-M-39 to CTX-M-41. Lastly, the fourth assay was conducted to detect minor ESBLs (VEB, PER, GES, ACC, MOX, and DHA). Additionally, we identified OXA β-lactamases (Groups OXA 1, 2, 51, and 58) using the primers previously described (Voets et al., 2011). For all samples, PCR was performed using Multiplex PCR Master containing Hot Start Taq polymerase (Jena Bioscience, Jena Germany) and 100 ng of DNA extracted from the samples, in a final volume of 25 µL. Amplification was carried out in a Cetus Thermal Cycler (Perkin-Elmer, Ueberlingen, Germany) under the specific conditions for each resistance gene to be detected. Amplified DNA fragments were separated on 1.5% agarose gels with 0.5X TBE (Tris-Borate-EDTA) buffer containing ethidium bromide (1 mg/ml). Gels were visualized using an iBright CL1000 imaging system (Invitrogen, Thermo Fisher Scientific). As a reference, DNA samples of previously reported isolates of *K. pneumoniae* carrying *bla*_CTX-M-15_ (GenBank ID: AGE61862.1) and *bla*_TEM-1_ (GenBank ID: ALJ57215.1) were utilized (Alcántar-Curiel et al., 2023). A clinical isolate of *E. coli* was employed as a positive control for *bla*_OXA-1_ (GenBank ID: CP13738.1), while a clinical isolate of *K. pneumoniae* was utilized as a positive control for *bla*_SHV_ (GenBank ID: AWD75419.1). *E. coli* ATCC 25922 was employed as the negative control.

### Clonal relationship between colonizing and invasive strains

Genetic relationships were determined by pulsed-field gel electrophoresis (PFGE) (Swaminathan et al., 2001). Genomic DNA was prepared in 1.2% agarose blocks and enzymatically digested with *Xba*I at 37°C for 2.5 h. DNA fragments were separated using the CHEF-DR II system (Bio-Rad Laboratories) with 1.1% agarose (SeaKem Gold^®^) at 5.5 V/cm, an initial pulse of 3.5 s, a final pulse of 30 s, and 14°C for 22 h. After staining and washing, the gel was visualized using an iBright CL1000 imaging system (Invitrogen, Thermo Fisher Scientific). Generated electrophoretic patterns were analyzed through visual inspection, detecting the position and number of bands. A dendrogram was constructed using simple matching and the unweighted pair-group method with arithmetic mean (UPGMA) clustering method using NTSYSpc. 2.1 software (Rohlf, 1998).

### Prevalence and risk factors for ESBL-PEco and ESBL-PKpn colonization and occurrence

The prevalence of fecal colonization and invasive HAIs caused by ESBL-PEco or ESBL-PKpn was determined. To identify the risk factors for HAIs due to ESBL-PEco or ESBL-PKpn in patients colonized by these bacteria, the characteristics of colonized patients who developed HAIs (cases) were compared with those of colonized patients who did not develop HAIs due to ESBL-PEco or ESBL-PKpn (controls). Variables related to gestational weeks, birth weight, admission weight, previous hospitalization, prior antibiotic use, repeated antibiotic use, duration of antimicrobial treatment, early sepsis, invasive device use, and nutrition type were recorded. The comparison between groups in the case of variables on a continuous scale was carried out either using the Wilcoxon test for a distribution other than normal or the Student t-test for a normal distribution (McElduff et al., 2010). In the case of categorical variables, the chi-square and Fisher’s exact tests were used (Nowacki, 2017). The alpha significance level used in all statistical tests was 5%.

## Results

### Study population, identification of the bacterial isolates, incidence of ESBL-PEco and ESBL-PKpn carriage

During the study period, 61 patients were enrolled. However, 14 patients were excluded from the study as they provide only a single rectal sample and could not continue with the follow-up. Among the remaining 47 patients, a total of 203 rectal samples were collected. In 45 of these patients (95.7%) and in 166 samples (81.7%), we identified 126 *E. coli* isolates and 111 *K. pneumoniae*. Specifically, 99 (78.6%) were categorized as ESBL-PEco, and 97 (87.4%) as ESBL-PKpn originating from a total of 41 patients (38 patients with ESBL-PEco, 39 patients with ESBL-PKpn, and 35 patients with both bacteria).

Nine out of these 41 patients experienced HAIs (22%) during their hospitalization, with six cases attributed to ESBL-PEco and three to ESBL-PKpn. These infectious events resulted in the isolation of 15 and 4 strains of each species (respectively), derived from different culture samples.

The peak colonization by ESBL-PEco and/or ESBL-PKpn was detected during the second week of hospitalization with a total of 18/41 (44%) patients, and the highest number of HAIs was also observed during the second week of hospitalization, with 6/12 (50%) cases (**Supplementary Figure 1**).

### Demographical and clinical characteristics

**Figure 1:**
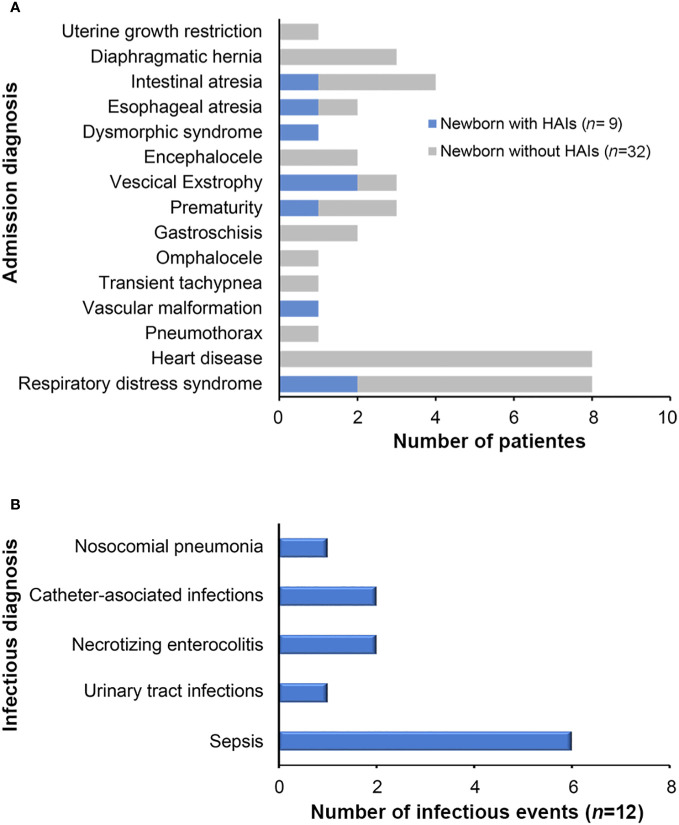
Diagnoses of neonatal patients colonized by ESBL-producing *Escherichia coli* and *Klebsiella pneumoniae*. **(A)** Admission diagnoses of patients colonized by ESBL-producing *Escherichia coli* and *Klebsiella pneumoniae* isolates who presented HAIs during hospitalization at the NICU. **(B)** HAIs diagnosed during the study period.

Slightly more of the colonized patients with ESBL-PEco and/or ESBL-PKpn were male 24/41 (58.5%), born by cesarean section 25 (60.9%), and premature born before 37 weeks of gestation 21/41 (51.2%), with an average weight at hospital admission of 2,076 g (SD ± 835.4, minimum 648 g, maximum 3,490 g). The use of antibiotics prior to admission was reported in 28 patients (68.2%), same as hospital unit, 28 patients (68.2%) required antibiotics, with an average duration of treatment of 7.5 days (SD± 8.7, minimum 5, maximum 45 days) (**Table 1**). The relationship between the admission diagnosis of patients colonized with ESBL-PEco and/or ESBL-PKpn and the progression of ESBL-PEco or ESBL-PKpn-HAIs is shown in **Figure 1A**.

**Table 1:** Descriptive statistics of the risk factors for the development of HAIs in colonized patients by ESBL-producing *Escherichia coli* or ESBL-producing *Klebsiella pneumoniae*.

Risk factors	Colonized patients with ESBL-producing *Escherichia coli* or ESBL-producing *Klebsiella pneumoniae* *n* = 41		
	Patients without HAIs Control group *n*= 32	Patients with HAIs Case group *n* = 9	*p*
**Sex** Male *n* (%) Female *n* (%)	19 (59.4) 13 (40.6)	5 (55.6) 4 (44.4)	0.84
**Delivery practice** Cesarean *n* (%) Vaginal *n* (%)	19 (59.4) 13 (40.6)	6 (66.7) 3 (33.3)	0.69
**Gestational age (weeks), mean (SD)** < 37 *n* (%) ≥ 37 *n* (%)	35.2 (4.6) 16 (50) 16 (50)	34 (4.8) 5 (55.6) 4 (44.4)	0.33 0.77
**Birth weight (g), mean (SD)**	2,196.5 (860.3)	2,083.3 (1,157.4)	0.75
**Weight at hospital admission (g), mean (SD)**	2,097 (788.1)	2,001.2 (1,036.6)	0.72
**Previous hospitalization *n* (%)**	22 (68.7)	8 (21.4)	0.40
**Use of antibiotics in the hospital unit *n* (%)**	20 (62.5)	8 (88.9)	0.23
**Hospital stay (days), mean ± SD**	30.7 ± 21	50.7 ± 29	0.03
**Repeated use of antibiotics *n* (%)**	3 (9.4)	3 (33.3)	0.11
**Duration of antibiotic treatment (days), mean (SD)**	4.8 (5.7)	17 (10.9)	<0.001
**Use of invasive devices (days), mean (SD) ** Catheter Orotracheal tube Chest tube Urinary catheter	9.2 (5.6) 11.8 (7.3) 1.8 (5.8) 0.22 (0.49)	23.8 (17.3) 25.2 (19) 2.3 (3.7) 5.4 (9.5)	0.002 0.004 0.57 0.01
**Type of nutrition (days), mean (SD) ** Fasting Enteral nutrition Parenteral nutrition	4.8 (7.3) 26.4 (20.6) 5.8 (7.6)	6.5 (6.7) 31.3 (23.5) 14.7 (24.3)	0.15 0.67 0.43

Upon admission of the newborns to the NICU, clinical data indicated suspicion of early sepsis in 16/47 (34%) cases, but none was corroborated by cultures. Throughout the follow-up period, after 48 hours of admission, 12 HAIs were clinically diagnosed in 41 of the patients included in the study, representing a prevalence of 29.3%. The types and frequency of HAIs are shown in **Figure 1B**, the most common infectious diagnosis was nosocomial sepsis with 6 cases (50%).

### Antimicrobial resistance pattern of colonizing and infectious ESBL-PEco and ESBL-PKpn

**Figure 2:**
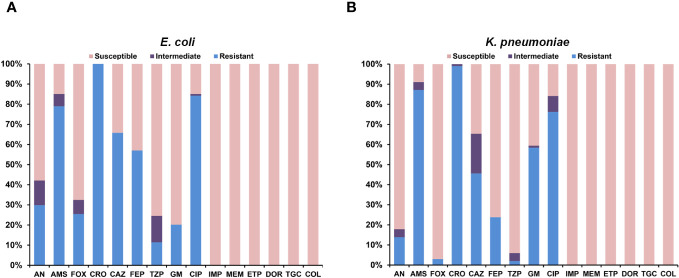
Antimicrobial susceptibility of colonizing and infectious ESBL-producing *Escherichia coli* and *Klebsiella pneumoniae.* **(A)** Isolates of *Escherichia coli*. **(B)** Isolates of *Klebsiella pneumoniae*.

All colonizing and infectious strains of ESBL-PEco (114 strains; 99 colonization strains and 15 HAIs strains) and ESBL-PKpn (101 strains; 97 colonization strains and 4 HAIs strains) were analyzed. One hundred percent of these strains were sensitive to carbapenems, tigecycline, and colistin. ESBL-PEco strains showed resistance rates of 25.4%, 100%, 65.7%, and 57.1% to cefoxitin, ceftriaxone, ceftazidime, and cefepime, respectively (**Figure 2A**), while in ESBL-PKpn strains, the resistance to the same cephalosporins was 2.9%, 99%, 45.5% and 23.7% (**Figure 2B**).

### Occurrence of ESBL genes

The *bla*_CTX-M-1 group_ gene was the most prevalent for both species, with 88/114 (77.2%) in ESBL-PEco and 83/101 (82.2%) in ESBL-PKpn, followed by *bla*_TEM_ 57/114 (50%) and *bla*_OXA-1_ 50/114 (43.86%) in ESBL-PEco, and *bla*_TEM_ 81/101 (80.2%) and *bla*_SHV_ 77/101 (76.2%) in ESBL-PKpn (**Table 2**). The majority of isolates, 203/215 (94.4%), showed the coexistence of at least two of the analyzed genes. In total, 11 different profiles were detected in ESBL-PEco and 19 in ESBL-PKpn, with TEM+CTX-M-1 group being the most frequent profile in the ESBL-PEco strains (35/114, 30.7%). Meanwhile, the TEM + SHV + CTX-M-1 group + OXA-1 profile was the most common profile in ESBL-PKpn (46/101, 45.5%). The maximum number of different types of ESBL-encoding genes detected in a single strain was five, observed in one strain of ESBL-PKpn (**Tables 3A, B**).

**Table 2:** Frequency of genes encoding resistance mechanisms in ESBL-producing *Escherichia coli* and *Klebsiella pneumoniae* isolated from neonates in the HIMFG NICU.

Gene	ESBL-PEco *n* = 114	%	ESBL-PKpn *n* = 101	%
***bla*_CTX-M-1 group_**	88	77.19	83	82.19
***bla*_TEM_**	57	50.00	81	80.19
***bla*_SHV_**	3	2.63	77	76.24
***bla*_OXA-1_**	50	43.86	58	57.43
***bla*_CTX-M-9 group_**	2	1.75	7	6.93

Table 3ADistribution of ESBL gene profiles among ESBL-producing *Escherichia coli* from neonates in the HIMFG NICU.

**Table:** 

ESBL profile (*n* = 114)	No isolates (%)	Number of genes in co-existence
***TEM + CTX-M-1 group***	35 (30.7)	2
***CTX-M-1 group + OXA-1***	24 (21)	2
***CTX-M-1 group***	19 (16.6)	1
***OXA-1***	11 (9.6)	3
***TEM + CTX-M-1 group + OXA-1***	7 (6.1)	2
***TEM + OXA-1***	7 (6.1)	1
***TEM***	4 (3.5)	1
***TEM + CTX-M-9 group***	2 (1.8)	2
***TEM + SHV + CTX-M-1 group + OXA-1***	1 (0.87)	4
***TEM + SHV + CTX-M-1 group***	1 (0.87)	3
***TEM***	1 (0.87)	1
***No gene detected***	2 (1.8)	0

**Table 3B:** Distribution of ESBL gene profiles among ESBL-producing *Klebsiella pneumoniae* from neonates in the HIMFG NICU.

ESBL profile (*n* = 101)	No isolates (%)	Number of genes in co-existence
***TEM + SHV + CTX-M-1 group + OXA-1***	46 (45.54)	4
***TEM + SHV + CTX-M-1 group***	10 (9.9)	3
***SHV + CTX-M-1 group***	6 (5.94)	2
***TEM + CTX-M-1 group***	6 (5.94)	2
***TEM + SHV + OXA-1***	6 (5.94)	3
***CTX-M-1 group***	5 (4.95)	1
***SHV***	4 (3.96)	1
***TEM + CTX-M-1 group + OXA-1***	3 (2.97)	3
***SHV + CTX-M-1 group + OXA-1***	2 (1.98)	3
***TEM***	2 (1.98)	1
***TEM + CTX-M-9 group***	2 (1.98)	2
***TEM + SHV***	2 (1.98)	2
***OXA-1***	1 (0.99)	1
***CTX-M-1 group + OXA-1***	1 (0.99)	2
***SHV + CTX-M-1 group + CTX-M-9 group***	1 (0.99)	3
***TEM + CTX-M-1 group + CTX-M-9 group***	1 (0.99)	3
***TEM + SHV + CTX-M-1 group + CTX-M-9 group***	1 (0.99)	4
***TEM + SHV + CTX-M-9 group***	1 (0.99)	3
***TEM + SHV + CTX-M-1 group + CTX-M-9 group + OXA-1***	1 (0.99)	5

### Clonality between colonizing and infecting ESBL-PEco and ESBL-PKpn isolates

**Figure 3:**
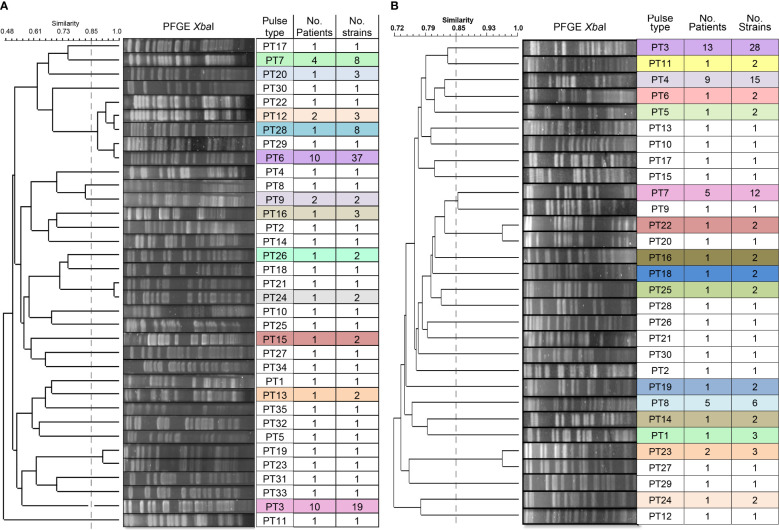
Dendrogram of the PFGE profiles of ESBL-producing *Escherichia coli* and *Klebsiella pneumoniae* isolates for colonization and HAIs in neonates hospitalized at the NICU. **(A)** A total of 114 isolates of *Escherichia coli* were grouped into 35 pulse types. **(B)** A total of 101 isolates of *Klebsiella pneumoniae* were grouped into 30 pulse types. The dashed line represents the 85% similarity level used in the cluster designation.

The determination of the genomic fingerprint of the 114 ESBL-PEco isolates detected 35 different pulse types, named in numerical order preceded by the initials PT (**Figure 3A**). Meanwhile, in the 101 ESBL-PKpn isolates, 30 different pulse types were detected, also named in numerical order preceded by the initials PT (**Figure 3B**).

**Figure 4:**
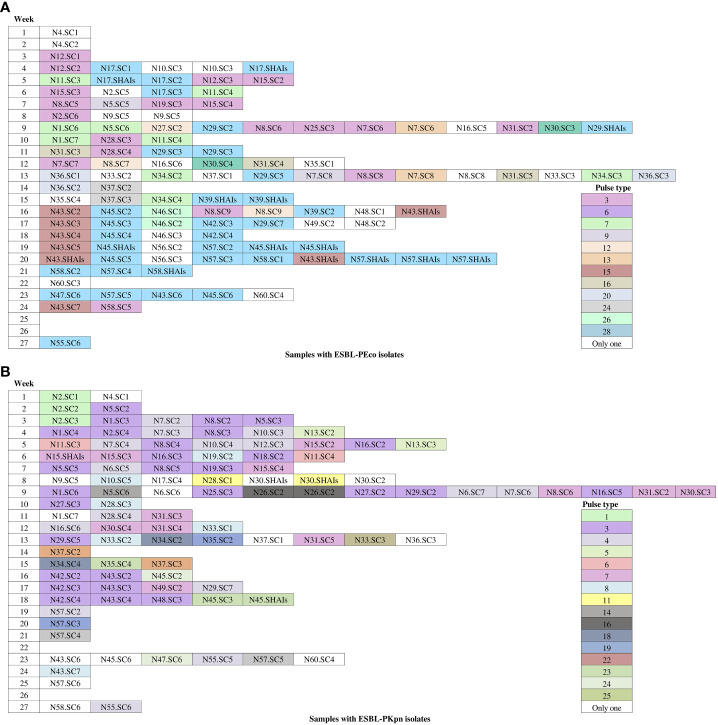
Occurrence of pulse types of ESBL-producing *Escherichia coli* and *Klebsiella pneumoniae* observed during the follow-up period. White-colored boxes represent different pulse types, each containing a single isolate for colonization and HAIs throughout the follow-up time. **(A)** *Escherichia coli* isolates. **(B)** *Klebsiella pneumoniae* isolates. N, neonate; SC, sample colonization; SHAIs, samples of HAIs.

We identified that 23 of 35 (65.7%) pulse types were detected once in ESBL-PEco strains, while the rest were found at least twice in the same or different patients. Is distinguished PT6 because was detected in 37 isolates from colonization samples out of ten patients, and five of them (N29, N39, N45, N57, and N58) progressed to HAIs, while PT3 was identified in 19 strains from 10 patients, and none of them developed an HAI related to this pulse type. Week 13 of the study stands out, during which 11 different pulse types were isolated in the NICU (**Figure 4A**).

In relation to ESBL-PKpn, we observed that PT3 was the most prevalent in the NICU. This pulse type 3 was detected sequentially in patients N5, N1, N8, N2, N16, N19, N25, N27, N29, N42, N43, and N48. None of them developed HAI. Once again, week 13 of the study stands out, where eight different ESBL-PKpn pulse types were detected in the NICU neonatology hospitalization area (**Figure 4B**).

**Figure 5:**
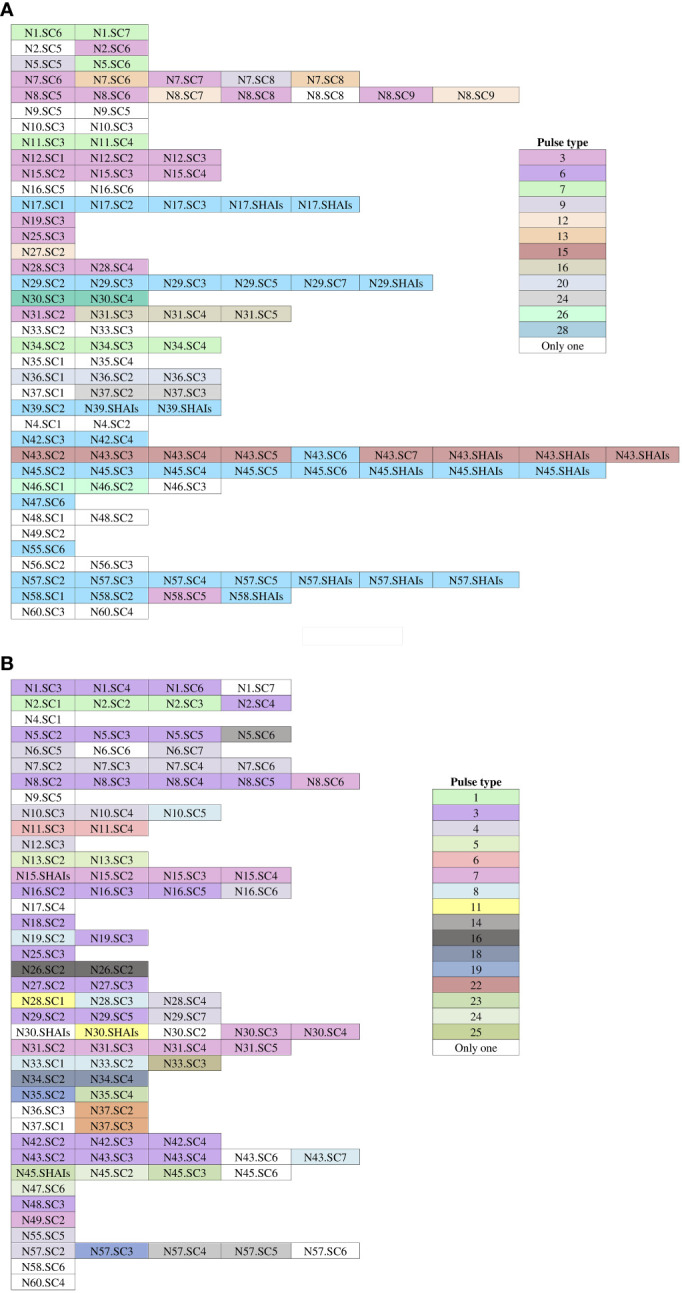
Pulse types of ESBL-producing *Escherichia coli* and *Klebsiella pneumoniae* from rectal samples collected from neonates during the follow-up time. **(A)** Isolates of *Escherichia coli.* **(B)** Isolates of *Klebsiella pneumoniae.* White-colored boxes represent different pulse types, each containing a single isolate. N, neonate; SC, sample colonization; SHAIs, sample of HAIs.

The analysis of the pulse types identified for each newborn is depicted in **Figure 5**. Our findings revealed that out of the 32 patients with ESBL-PEco present at least in two study time points, 21 (65.6%) demonstrated the same pulse types, suggesting a colonization event. Conversely, 11 out of 32 patients (34.4%) showed different ESBL-PEco pulse types at different time points, suggesting a transient acquisition of these clones. Similarly, among the 26 patients with ESBL-PKpn present at least in two study time points, 23 (88.5%) exhibited the same pulse types, suggesting a colonization event. In contrast, 3 out of 26 patients (11.5%) displayed unique ESBL-PKpn pulse types at different time points, suggesting a transient acquisition of these clones. Finally, clonality analysis revealed that five of seven patients colonized with ESBL-PEco (71.4%) and two of three patients colonized with ESBL-PKpn (66%) progressed to HAIs with the same colonizing isolate.

### Risk factors for the occurrence of ESBL-PEco and ESBL-PKpn-HAIs

The analysis of clinical characteristics in the group of patients colonized by ESBL-PEco or ESBL-PKpn who did not develop HAIs (controls) and those who did progress to ESBL-PEco or ESBL-PKpn -HAIs (cases), revealed that variables associated with a higher risk of progression to ESBL-PEco and ESBL-PKpn-HAIs were hospital-length of stay (p = 0.03), duration of antibiotic treatment (p= <0.001) (**Table 1**), and the use of invasive devices such as catheters, (p= 0.002), orotracheal (p=0.004), and urinary catheters (p=0.01) (**Table 1**). There were two deaths in the group of ESBL-PEco and ESBL-PKpn-HAIs, representing 0.17% of the total patients in that group, and no differences were found between the groups with and without ESBL-PEco or ESBL-PKpn-HAIs.

## Discussion

In this prospective study, it was revealed that upon admission to the HIMFG NICU, 27.6% (13/47) of patients were already colonized by ESBL-PEco and ESBL-PKpn. The percentage increased to 87% (41/47) by the end of the follow-up period, exceeding the reporting range, which typically ranged from 8% to 83% (Huerta-García et al., 2015; Folgori et al., 2018; Hagel et al., 2019; Edwards et al., 2023). Notably, the study by Huerta-García et al. carried out in our country reported that during the second week of hospital stay, 81% of neonates were intestinally colonized by ESBL-PEco and ESBL-PKpn, while our study revealed 78.7% (37/47) colonization during the same week of hospitalization (Huerta-García et al., 2015). This demonstrates the rapid and dynamic nature of the colonization process, which could be influenced by various previously reported factors (Martin and Bachman, 2018; Yin et al., 2021). These factors encompass prolonged hospital stays, often resulting in increased utilization of invasive techniques. Additionally, a significant majority of these patients receive antimicrobial treatments, exerting selective pressure on these pathogens. Horizontal colonization via objects used in patient care, combined with inadequate hand hygiene practices among healthcare workers responsible for their care, represents another potential contributing factor. Moreover, insufficient nursing staff levels might hinder the delivery of adequate care to these patients.

As observed in this study, of the 41 patients colonized with ESBL- ESBL-PEco or ESBL-PKpn, 9 (22%) developed HAIs during their hospital stay in the NICU, representing a high infection rate compared to a previous report conducted in 2020 by Denkel et al., (Denkel et al., 2020) where they observed that ICUs exhibited the highest infection rate (17.2%), with 5.6% of HAIs attributed to ESBL-EP colonization. They also found that in 98% of patients with HAIs, the colonizing isolate and the pathogenic agent responsible for the HAI exhibited genetic identity (Denkel et al., 2020). Our results demonstrated that in some of the patients, colonization, and infection occurred with a bacterial strain of the same pulse type. Although the number of patients in this study is small, it supports the hypothesis that many infections, including bacteremia, originate from an intestinal reservoir (Denkel et al., 2014; Zerr et al., 2014; Platteel et al., 2015; Gorrie et al., 2017; Armand-Lefèvre et al., 2018; Souverein et al., 2019; Campos-Madueno et al., 2023).

The main risk factors observed development of HAIs include the duration of hospitalization, prior antibiotic use in the NICU, the length of antibiotic treatment, and the extended use of invasive devices such as orotracheal tubes and venous catheters. Our findings align with a study conducted by Smith et al., in 2010 (Smith et al., 2010), which identified increased use of central venous catheters and mechanical ventilation as risk factors for colonization and bloodstream infections by gram-negative bacilli, reflecting our study’s observation regarding the duration of orotracheal tube use (Smith et al., 2010). Other risk factors identified in previous studies, such as mode of delivery, did not yield significant values as risk factors in our study (Reyman et al., 2019; Pirr and Viemann, 2020).

In the current study, we found that ESBL-PEco and ESBL-PKpn isolates, both colonizers and causative agents of HAIs, exhibited 100% susceptibility to carbapenems, tigecycline, and colistin. This keeps them within the appropriate therapeutic options for HAIs. Of concern is the resistance observed with cefepime (ESBL-PEco: 57.1%, ESBL-PKpn: 29.8%), which is the first-line empirical antibiotic choice for HAI treatment at our institution. As our results demonstrate, in the case of ESBL-PEco, there is less than a 50% possibility that treatment will be adequate before de-escalation therapy.

Currently, the rapid spread of ESBL-producing bacteria has led to their widespread distribution throughout the world. Several studies identify *E. coli* and *K. pneumoniae* as the main ESBL producers (Obeng-Nkrumah et al., 2013; Ouedraogo et al., 2016; Siriphap et al., 2022).

Although SHV and TEM were the first beta-lactamases to spread, they have gradually been replaced by CTX-M types. This phenomenon has been associated with the high mobilization of the genes encoding them (Shahid et al., 2011). The present study identified the CTX-M-1 group as the most common beta-lactamase gene in ESBL-PEco strains (77%) and in ESBL-PKpn (82.19%). These results are consistent with those reported in Germany (Schmiedel et al., 2014) and Ethiopia (Tufa et al., 2020), where the prevalence of CTX-M in both species was 50% and 82.7% respectively, while in India (Shahid et al., 2011), Romania (Ghenea et al., 2022), and Nepal (Koirala et al., 2021), ESBL-PEco strains showed 4.5%, 92.8 and 93.8% respectively and ESBL-PKpn strains, 39.2%, 71.8%, and 78.9%. Among our strains, CTX-M-9 showed a low prevalence (1.75% and 6.93% for ESBL-PEco and ESBL-PKpn, respectively). The low frequency of this beta-lactamase in Mexico can be attributed to its geographical origin so far our country, since it emerged in Lithuania, between 2012 and 2014. The authors reported its presence in 90.6% of bloodstream isolates of ESBL-PEco (Kirtikliene et al., 2022). This geographical origin affects its distribution, and the limited frequency suggests it may have difficulty spreading among different bacteria in the Mexican environment, possibly due to unique genetic traits or limited opportunities for dissemination.

The simultaneous occurrence of genes encoding distinct beta-lactamases has garnered attention in contemporary research, with numerous studies highlighting a progressive increase in prevalence over time (Munday et al., 2004; Oteo et al., 2010; Shahid et al., 2011; Schmiedel et al., 2014). Our investigation revealed an overwhelming 98% of isolates exhibited the coexistence of at least two distinct beta-lactamase genes. This percentage surpasses the reported frequencies in Iran (Rezai et al., 2015), Malaysia (Mahdi Yahya Mohsen et al., 2016), India (Bora et al., 2014), and Germany (Schmiedel et al., 2014), standing at 32%, 78%, 57%, and 78%, respectively. In this intricate landscape, a study conducted in a Bosnian and Herzegovinian hospital unveiled that a substantial 77.3% of isolates from hospitalized patients harbored more than two distinct ESBL types (Ibrahimagić et al., 2017). Notably, the TEM + SHV + CTX-M-1 group + OXA-1 profile dominated, prevailing in ESBL-PKpn strains (63%, 28/44) and ESBL-PEco strains (10%, 1/10). Surprisingly, this specific ESBL profile is the same predominant pattern we identified in our research, manifesting in ESBL-PKpn strains (45.54%, 46/101) and an isolated ESBL-PEco strain (0.88%, 1/114). This compelling discovery underscores the successful coexistence of these resistance genes in ESBL-PKpn and alludes to the possibility of their dissemination via a plasmid, consistent with previous research (Cantón et al., 2012).

The clonality analysis performed highlighted the dissemination of clonal strains to other patients during the follow-up period. For instance, PT6 of ESBL-PEco was isolated in the initial rectal sample from patient N17.SC1. Three days later, due to symptoms, blood and urine samples were collected, and PT6 was found in both samples. Subsequently, PT6 was detected in colonization samples from nine patients, and in five of them (N29, N39, N45, N57, and N58), this pulse type progressed to HAIs. It is worth noting that in the subsequent rectal samplings of these patients, PT6 continued to be detected. At the end of the study, 37 PT6 strains were isolated in these 10 patients, one of whom (patient N43) developed HAIs related to PT28 (which was not detected in any other patient) and also showed colonization by PT6 in the sixth rectal sample. Another example was PT3 was detected upon admission (first rectal sample) in patient N12.SC1. Subsequently, in the fifth week of the study, it was identified in patient N15. Two weeks later, it was found in two other patients (patients N19 and N8), and it continued to disseminate until the end of the study. In total, we identified 19 PT3 strains in 10 patients, and none of them developed an HAI related to this pulse type. The 13th week of the study stands out, during which 11 different pulse types were isolated in the NICU (**Figure 4A**).

About ESBL-PKpn, we observed that PT3 was prevalent in the NICU. This pulse type was initially identified during the second week of patient´s N5 admission. Subsequently, it was sequentially spread to patients N1, N8, N2, N16, N19, N25, N27, N29, N42, N43, and N48. None of the 12 patients developed HAIs. Notably, patient N15, in whom PT7 was identified upon admission and during the first week of hospitalization, subsequently developed an HAI caused by the same clone. In contrast, patient N30 experienced a HAI that was not related to the previously isolated pulse types in their rectal samples. A particular case was patient N45, who was colonized with ESBL-PEco PT6 (associated with HAIs in other patients) starting from the second week with ESBL-PKpn PT24 and in the fourth week for ESBL-PKpn PT23. After six days, this patient developed HAIs in which ESBL-PEco PT6 and ESBL-PKpn PT23 were identified. Once again, week 13 of the study stands out, where eight different *K. pneumoniae* pulse types were detected in the NICU (**Figure 4B**).

We did not consider the progression to infection in patient N43 because *E. coli* P28 was detected both in the admission rectal sample and in the HAIs samples taken on the same day. We also excluded patient 39, whose initial rectal sample showed no bacterial growth, but on the following day of hospitalization, they were diagnosed with HAIs, confirmed by a positive culture for *E. coli* PT6. Their second colonization sample also belonged to the same pulse type.

The clonality analysis carried out on both colonization and infection isolates from the same patients showed that colonization preceded infection in 70% of cases. These findings underscore the frequent occurrence of cross-transmission events within our hospital’s NICU. Consequently, continuous surveillance in this clinical setting is imperative, with particular emphasis on optimizing patient management to mitigate the substantial clonal dissemination and, subsequently, reduce the risk of HAIs.

Over the past decade, there has been a growing body of research aimed at elucidating the relationship between microbial colonization and the development of HAIs. As a result, we now better understand several factors that can disrupt the composition of the microbiome. These factors encompass variables such as the mode of birth, antibiotic prophylaxis administered to mothers undergoing cesarean sections, and antibiotic treatments (Vandenplas et al., 2020; Socha-Banasiak et al., 2021). Additionally, several hypotheses have emerged, including one suggesting that infections in colonized neonates may be attributed to the translocation of bacteria from the gastrointestinal tract to the bloodstream, facilitated by an immature or compromised intestinal barrier. Furthermore, indirect transmission through alternative routes due to the immaturity of defense mechanisms has been postulated (Basu, 2015). Collectively, these factors underscore the vulnerability of this neonatal population and emphasize the need for comprehensive research and intervention efforts.

One of the limitations encountered in this study was the relatively modest sample size of patients included, a consequence of the SARS-CoV-2 pandemic, which imposed restrictions on patient enrollment. Additionally, the confinement of the study to a single hospital setting is another limitation to consider. Consequently, it is important to acknowledge that the results obtained may not be fully representative of patients in the broader community or in other urban regions of Mexico. Nevertheless, despite these limitations, the study findings offer valuable insights and support the recommendation for the implementation of early ESBL-PEco and ESBL-PKpn identification strategies, and appropriate infection control and practices. These proactive measures have the potential to reduce the notable rates of bacterial colonization observed, consequently mitigating the risk of bacterial dissemination and HAIs.

## Conclusions

This investigation has revealed that intestinal colonization by ESBL-PEco and ESBL-PKpn is a prevalent occurrence in hospitalized newborns, indicating that a considerable portion of them are susceptible to progressing to HAIs. Furthermore, patients colonized by ESBL-PEco and ESBL-PKpn strains producing CTX-M and TEM, deserve further attention due to the observed association between the development of HAIs and factors such as prolonged hospitalization and the use of invasive medical devices. The genetic similarity observed between colonizing and infecting ESBL-PEco and ESBL-PKpn strains underscores the importance of endogenous infection and clonal dissemination as predominant contributors to HAIs, predominantly attributed to *E. coli*. This study provides valuable insights that improve our understanding of the epidemiology of ESBL-producing *Escherichia coli* and *Klebsiella pneumoniae* and intestinal colonization in neonatal patients.

## Data availability statement

The raw data supporting the conclusions of this article will be made available by the authors, without undue reservation.

## Ethics statement

The studies involving humans were approved by the Research, Ethics, and Biosafety Committees. Hospital Infantil de México Federico Gómez. HIM/2018/073. The studies were conducted in accordance with the local legislation and institutional requirements. Written informed consent for participation in this study was provided by the participants’ legal guardians/next of kin.

## Author contributions

VJ-R: Writing – review & editing, Conceptualization, Funding acquisition, Investigation, Supervision, Writing – original draft. DV-G: Conceptualization, Writing – review & editing. AM-V: Investigation, Writing – original draft. RA-V: Writing – original draft, Methodology. PA-R: Methodology, Writing – original draft. BL-M: Methodology, Writing – original draft. AR-L: Methodology, Writing – original draft. MA-C: Conceptualization, Writing – review & editing.

## Supplementary material

Supplementary Figure 1 Frequency of colonization of ESBL-producing *Escherichia coli* and *Klebsiella pneumoniae* isolates for colonization and HAIs during the weeks of hospitalization of neonates admitted at the NICU.
