# Supplementary figures and images for: Gut colonization and subsequent infection of neonates caused by extended-spectrum beta-lactamase-producing Escherichia coli and Klebsiella pneumoniae
Source: Front Cell Infect Microbiol. 2024 Jan 19;13:1322874. doi: 10.3389/fcimb.2023.1322874 (PMC10834783; doi:10.3389/fcimb.2023.1322874)

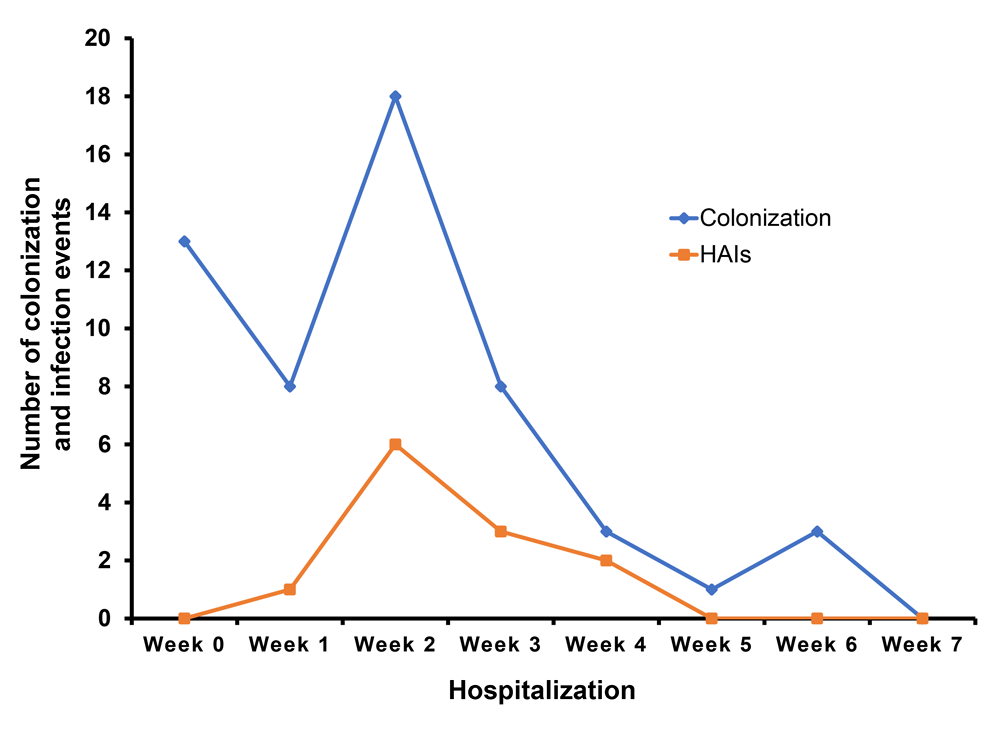

Supplement: Supplementary Figure 1 — Frequency of colonization of ESBL-producing Escherichia coli and Klebsiella pneumoniae isolates for colonization and HAIs during the weeks of hospitalization of neonates admitted at the NICU. [file Image_1.tif]
